# Digital Cognitive Behavioral Therapy (CBT) Apps for ADHD: A Systematic Review of Recent Evidence

**DOI:** 10.1192/j.eurpsy.2025.1417

**Published:** 2025-08-26

**Authors:** A. Alalsultan Alghory

**Affiliations:** 1 Istanbul Medipol University, Istanbul, Türkiye

## Abstract

**Introduction:**

Attention-deficit/hyperactivity disorder (ADHD) is a neurodevelopmental condition characterized by persistent patterns of inattention, hyperactivity, and impulsivity. Cognitive Behavioral Therapy (CBT) is a well-established intervention for ADHD, helping individuals manage symptoms by targeting dysfunctional thought patterns and behaviors. In recent years, the accessibility of digital CBT apps has expanded, providing a potential alternative to traditional therapy. This systematic review aims to assess the efficacy of digital CBT apps for symptom management in individuals with ADHD.

**Objectives:**

This review seeks to (1) evaluate the effectiveness of digital CBT apps in reducing ADHD-related symptoms such as inattention and impulsivity, (2) assess the adherence and usability of these apps, and (3) identify gaps in the literature to guide future research on digital interventions for ADHD.

**Methods:**

A systematic search was conducted using PubMed and Google Scholar to identify peer-reviewed studies published within the last 5 years (2019–2024). The search terms used included “ADHD,” “digital CBT,” “mobile apps,” and “cognitive behavioral therapy.” Inclusion criteria were studies that focused on the use of digital CBT interventions specifically for ADHD symptom management in both children and adults. Studies that assessed the effectiveness of these interventions using standardized symptom rating scales were prioritized. Articles that involved non-CBT digital interventions, case reports, or review articles were excluded.

**Results:**

Six studies met the inclusion criteria. The results showed promising outcomes for ADHD symptom reduction, particularly in reducing inattention and impulsivity. Lenhard et al. (2018) demonstrated significant improvement in ADHD symptoms in children using digital CBT programs. Nigg et al. (2020) found similar improvements in adults. Sibley et al. (2021) observed enhanced executive functioning with high adherence to app-based interventions. In Torous et al. (2020), digital CBT apps improved patient engagement and accessibility to care. Hirvikoski et al. (2017) reported moderate effectiveness in symptom control but emphasized the need for continued monitoring of user adherence. Russell et al. (2021) highlighted the potential for personalization in digital therapies, although further research is needed to optimize these interventions.

**Conclusions:**

The review suggests that digital CBT apps offer a promising approach for ADHD symptom management, particularly in increasing accessibility and adherence to therapy. While these interventions show moderate to significant effectiveness in reducing core symptoms like inattention and impulsivity, further research is needed to address personalization and long-term efficacy. This highlights the growing potential for digital mental health interventions to complement traditional ADHD treatments.

**Disclosure of Interest:**

None Declared

